# Anti-Tumor Activity of a Novel Protein Obtained from Tartary Buckwheat

**DOI:** 10.3390/ijms11125201

**Published:** 2010-12-17

**Authors:** Xiaona Guo, Kexue Zhu, Hui Zhang, Huiyuan Yao

**Affiliations:** 1 State Key Laboratory of Food Science and Technology, Jiangnan University, 1800 Lihu Road, Wuxi-214122, Jiangsu, China; E-Mails: zkx1978@hotmail.com (K.Z.); hui1003@hotmail.com (H.Z.); hyyao@jiangnan.edu.cn (H.Y.); 2 School of Food Science and Technology, Jiangnan University, 1800 Lihu Road, Wuxi 214122, Jiangsu, China

**Keywords:** tartary buckwheat, protein, breast cancer cells, apoptosis, antitumor

## Abstract

TBWSP31 is a novel antitumor protein that was isolated from tartary buckwheat water-soluble extracts. The objective of this paper was to investigate the anti-proliferative effects of TBWSP31 on breast cancer Bcap37cells and to explore its possible mechanism. After treatment of Bcap37 cells with TBWSP31, typical apoptotic morphological changes were observed by inverted microscopy and scanning electron microscopy (SEM), such as detachment from the culture plate, change to a round shape, cell shrinkage, the absence of obvious microvilli, plasma membrane blebbing, and formation of apoptotic bodies. Cell-cycle analysis revealed that treatment with TBWSP31 resulted in a G_0_/G_1_ arrest and prevented the cells from growing from G_0_/G_1_ phase to S phase, which was most prominent at 48 h. The expression of bcl-2 and Fas were detected quantitatively by FCM, which showed that TBWSP31 induced-apoptosis may be involved with the participation of Fas and bcl-2. These results suggest that TBWSP31 is a potential antitumor compound and that apoptosis induced by TBWSP31 is a key antitumor mechanism.

## 1. Introduction

The genus *Fagopyrum* has about 15 species distributed in different parts of the world [1]. Among these species, only two types of buckwheat are used as food around the world: common buckwheat (*Fagopyrum esculentum* Moench) and tartary buckwheat (*Fagopyrum tataricum* Gaertn.) [2].

Tartary buckwheat grain, as an important functional food material, is commonly taken in the diet in eastern Asian countries [3]. It is well known that tartary buckwheat contains higher rutin and vitamins B1, B2, and B6 content than common buckwheat [4,5]. In China, tartary buckwheat is mainly grown in some mountainous regions, such as Liang Shan Yi Autonomous region in Sichuan province and Jing Zhou in Gui Zhou province [6].

Buckwheat protein has high biological value due to a well-balanced amino acid pattern and is rich in lysine and arginine. Recently, the physiological properties of buckwheat protein have also been studied. In rat feeding experiments, studies have proven that buckwheat protein extract has hypocholesterolemic [7–10], anticonstipation [11] and antiobesity activities [12]. In addition, Liu *et al.* [13] reported that buckwheat protein product had a protective effect against 1,2-dimethyhydrazine (DMH)-induced colon carcinogenesis in rats by reducing cell proliferation. Kayashita *et al.* reported that consumption of the buckwheat protein extract retarded 7,12-dimetylbenz (α) anthracene (DMBA)-induced mammary carcinogenesis in rats [14].

A tumor is a disease state characterized by uncontrolled proliferation and a loss of apoptosis. Cell proliferation and death are involved in the maintenance of homeostasis in normal tissues, and cell death is often inhibited in tumors with a higher cell proliferation rate [15]. Apoptosis is the process of programmed cell death through a tightly controlled program that plays important roles in many normal processes, ranging from fetal development to adult tissue homeostasis [16]. Thus, modulating apoptosis may be useful in the management and therapy or prevention of cancer. Apoptotic induction has been a new target for innovative mechanism based drug discovery. Compounds that block or suppress the proliferation of tumor cells by inducing apoptosis are considered to have potential as antitumor agents [17]. Apoptosis and necrosis are two different types of cell death via distinct morphological and biochemical processes [18,19].

Breast cancer is the most common cancer affecting women worldwide. It accounts for approximately 25% of all female malignancies with a higher prevalence in developed countries. Incidence rates continue to increase especially in the USA, Canada, the Nordic countries, Singapore and Japan, with over one million new cases reported each year worldwide [20]. An attractive method for breast carcinoma chemotherapy is the use of pharmaceutical agents that induce death of breast tumor cells to overcome proliferation. It is important to screen apoptotic inducers from plants, either in the form of crude extracts or as components isolated from them.

In our previous study, the effective antitumor protein (coded as TBWSP31) was isolated and purified from Chinese tartary buckwheat water-soluble extracts, and the antitumor mechanism of TBWSP31 was not clearly elucidated [21]. The antitumor effect of TBWSP31 was determined by MTT assay and we tested a number of different cell lines, such as Bel-7402 (human liver cancer cells), SGC-7901 (human gastric cancer cells), MDA-MB-231 (human breast cancer cells), Bcap37 (human breast cancer cells), MCF-7 (human breast cancer cells) and KB (human mouth carcinoma cells). Of the cancer cells tested, the most sensitive tumor cell to TBWSP31 was the human mammary cancer cell (unpublished data). The main objective of this study was to investigate the mechanism underlying the anti-tumor effect of TBWSP31 on the human breast cancer cell line Bcap37 and to explore whether the effect is mediated via an apoptotic mechanism. In this paper, morphological changes of Bcap37 cells were observed by inverted microscopy and scanning electron microscopy (SEM). Furthermore, the cell cycle and the expression of bcl-2 and Fas were analyzed using flow cytometry.

## 2. Results and Discussion

### 2.1. Trypan Blue Exclusion Test

**Figure 1.**
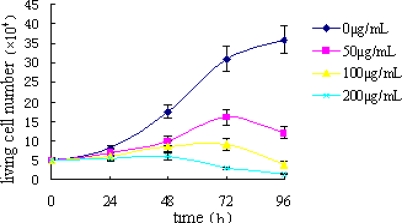
Effect of TBWSP31 treatment on Bcap37 cell growth. Bcap37 cells were treated with different concentration of TBWSP31 for 24, 48, 72 and 96 h. Data are shown as means ± SD (*n* = 4).

Figure 1 shows the effect of TBWSP31 treatment on Bcap37 cells as a growth curve. The dynamic change of Bcap37 cell proliferation in the presence of TBWSP31 is reflected by the cells’ growth curve. The effect of TBWSP31 treatment on Bcap37 cell growth was determined using the trypan blue exclusion test after 96 h of incubation. As shown in Figure 1, the number of living untreated Bcap37 cells increased linearly with the increase of incubation time. However, when Bcap37 cells were treated with 200 μg/mL of TBWSP31, the living cell number scarcely increased over the length of the experiment. The cells treated with 100 μg/mL of TBWSP31 showed slow growth and the cell growth curve reached a plateau before the incubation time reached 72 h. Furthermore, the number of living Bcap37 cells treated with 50 μg/mL increased until the incubation time of 72 h had been reached, and then decreased, which indicated that the inhibitory effect of TBWSP31 on Bcap37 cell proliferation was not due to direct killing of Bcap37 cells. In this part, we only investigated the effect of higher doses of TBWSP31 (50–200 μg/mL) on the growth of Bcap37 cells. The inhibition effect of lower concentrations of TBWSP31 (5–50 μg/mL) on the proliferation of Bcap37 cells was assessed by colorimetric MTT-assay in our previous study. For the rest of the current paper, lower concentrations of TBWSP31 were used because Flow cytometry (FCM) analysis needed a minimum amount of cells per experimental condition.

### 2.2. Morphological Observations by Inverted Microscopy

**Figure 2.**
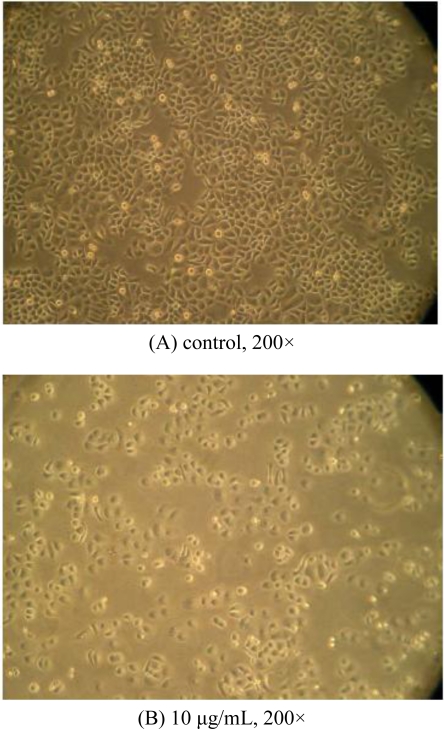
The TBWSP31-dependent morphology changes of Bcap37 cells observed under an inverted microscope. Bcap37 cells were cultured with (**A**) 0 μg/mL of TBWSP31 for 24 h; (**B**) Bcap37 cells were cultured with 10 μg/mL of TBWSP31 for 24 h.

Figure 2 shows the morphology changes of Bcap37 cells after treatment with TBWSP31 as observed by inverted microscopy. Bcap37 cells were cultured with 0 (control) or 10 μg/mL of TBWSP31 for 24 h. Under an inverted microscope, cell shape and changes can be clearly observed. As shown in Figure 2A, cells in the control group showed adherent growth and a regular polygon shape, with very few round cells. The growth speed of the control Bcap37 cells was higher than that of the TBWSP31-treated cells, indicating that TBWSP31 inhibited Bcap37 cell proliferation. Compared to the control, marked morphological changes were observed in the cells treated with 10 μg/mL of TBWSP31 for 24 h (Figure 2B). The adherent ability of Bcap37 cells became worse and the shape became round. Additionally, only few cells remained attached and the number of floating smaller cells increased in the medium after 24 h incubation with 10 μg/mL of TBWSP31, indicating the possibility of apoptosis occurrence [22].

### 2.3. Morphological Observation by SEM

**Figure 3.**
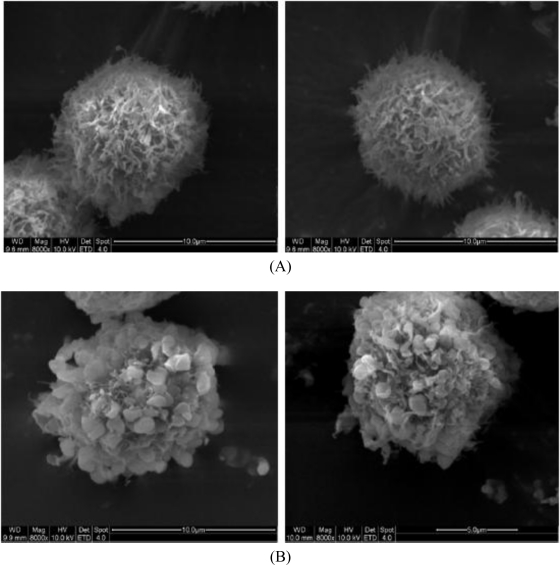
The morphological changes of Bcap37 cells as observed by scanning electron microscopy (SEM; 8000×). Two images of Bcap37 cells cultured with 0 μg/mL (**A**) or 20 μg/mL (**B**) of TBWSP31 for 48 h.

To further investigate whether TBWSP31 mediated cell death of Bcap37 cells is due to an apoptotic mechanism, the surface morphological changes that occurred with 20 μg/mL of TBWSP31 treatment for 48 h were observed under SEM. Figure 3 shows the morphological changes of Bcap37 cells observed by SEM.

The control group showed untreated cells with numerous microvilli over the surface of the cells and intact cells (Figure 3A). In contrast, cells treated with TBWSP31 exhibited a significant decrease in the number of microvilli and an apparent deformation (Figure 3B). The surface of Bcap37 cells became relatively smooth with no obvious microvilli, and some irregular blebs and apoptotic bodies were seen in cell membranes after treatment. Membrane blebbing is one of a series of distinctive morphological events during apoptosis. It has been reported to be associated with cytoplasmic and nuclear manifestations of apoptosis [23]. Highly conserved morphological changes, including membrane blebbing and cell shrinkage, have been used as morphological criteria to identify apoptotic cell death [24,25]. Cell shrinkage and plasma membrane blebbing could be observed after incubation of Bcap37 cells with TBWSP31. Treatment of Bcap37 cells with TBWSP31 resulted in significant morphological alterations, which are typical characteristics of apoptosis.

### 2.4. Cell Cycle Analysis

Table 1 shows the effect of TBWSP31 on the percentage of Bcap37cells present in each cell cycle phase (G_0_/G_1_, S, and G_2_/M). After 24 h incubation with 0 (control), 5 and 10 μg/mL of TBWSP31, the cell population in the G_0_/G_1_ phase was 53.6%, 57.3% and 68.4%, the cell population in S phase was 33.5%, 31.4% and 28.9%, and the cell population in G_2_/M phase was 12.9%, 11.3% and 2.7%, respectively. After 48 h incubation with 5 and 10 μg/mL of TBWSP31, the cell population in the G_0_/G_1_ phase increased to 70.9% and 77.6%, and the cell population in S phase decreased to 28.5% and 21.8%, respectively (*p* < 0.01). These results showed that TBWSP31 could inhibit the growth of Bcap37 cells through a cell cycle arrest in the G_0_/G_1_ phase, and prevent the cells growing from G_0_/G_1_ phase to S phase (*p* < 0.05). In addition, the inhibition effect of TBWSP31 on the proliferation of Bcap37 cells existed in a dose- and time-dependent manner.

### 2.5. Effect of TBWSP31 on Levels of bcl-2 and Fas Proteins in Bcap37 Cells

**Figure 4.**
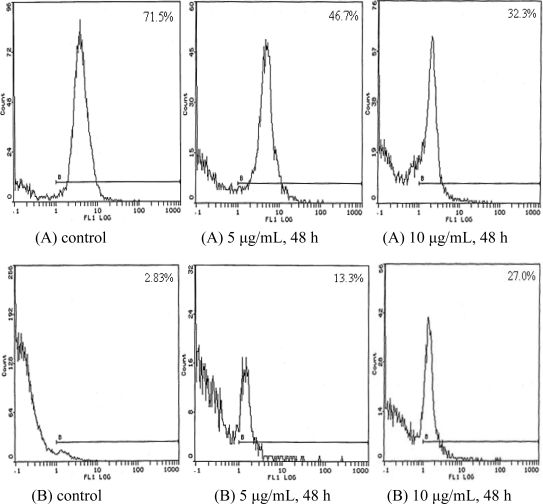
The alteration of bcl-2 (**A**) and Fas (**B**) protein levels of Bcap37 cells after treatment with TBWSP31, as determined by flow cytometry. Bcap37 cells were treated with 0 (control; left panels), 5 (middle panels) and 10 μg/mL (right panels) of TBWSP31 for 48 h. The percentage of the Bcl-2 and Fas protein are shown at the top right of each panel.

The effect of TBWSP31 on the levels of bcl-2 and Fas proteins in Bcap37 cells is shown in Figure 4. The level of bcl-2 decreased dramatically from 71.5% (0 μg/mL, control) to 46.7% and 32.3% when Bcap37 cells were treated with 5 μg/mL and 10 μg/mL of TBWSP31, respectively (*p* < 0.01) (see Figure 4A). In contrast, the levels of Fas protein increased significantly after TBWSP31 treatment: from 2.83% (0 μg/mL, control) to 13.3% (5 μg/mL) and 27.0% (10 μg/mL) (*p* < 0.01) (Figure 4B). These results showed that TBWSP31 treatment induced a significant decrease of bcl-2 expression and increase of Fas expression after 48 h in a dose-dependent manner. Cell apoptosis is regulated by anti-apoptotic and pro-apoptotic effectors, involving a large number of biomolecules. Bcl-2 is known to be an upstream effector molecule in the apoptotic pathway and is identified as a potent suppressor of apoptosis [26]. Fas protein, a glycosylated 45-kD transmembrane receptor, belongs to the tumor necrosis factor receptor family and induces apoptosis [27]. In the present study, the expression of bcl-2 was down-regulated and the expression of Fas was up-regulated. The changes of these proteins-correlated apoptosis expressions suggested that TBWSP31 induced-apoptosis may be involved with the participation of Fas and Bcl-2.

## 3. Experimental Section

### 3.1. Materials

Tartary buckwheat flour was obtained from the milling factory for minor crops in Liang Shan region in Sichuan province. Flour was defatted for 24 h with *n*-hexane under continuous stirring, air-dried at room temperature, and stored at 4 °C until used.

### 3.2. Preparation of TBWSP31

TBWSP31 was isolated from tartary buckwheat water-soluble extracts and purified by DEAE-Sepharose FF anion exchange, Sephadex G-100 gel filtration and Sephacryl S-200 gel filtration column chromatography.

### 3.3. Cell Culture

Human breast cancer (Bcap37) cell culture was maintained in RPMI-1640 medium (Gibco BRL, Grand Island, NY) supplemented with 10% fetal bovine serum at 37 °C in maximal humidity atmosphere containing 5% CO_2_.

### 3.4. Trypan Blue Exclusion Test

The effect of TBWSP31 treatment on Bcap37 cells growth was determined by the trypan blue exclusion test [28]. Bcap37 cells were plated in 24-well culture plates at 5 × 10^4^ cells/well and were treated with serial dilutions of TBWSP31 in quadruplicate. Wells containing different dilutions were selected to be counted every 24 h and cells that excluded trypan blue were counted in a Neubauer chamber. The growth curve of Bcap37 cells was drawn according to their average value.

### 3.5. Light Microscopy

Bcap37 cells were seeded in RPMI-1640 containing 10% FBS and treated with 0 (control) and 10 μg/mL of TBWSP31 for 24 h. The cellular morphology was directly observed using an inverted microscope at the indicated times.

### 3.6. SEM

Bcap37 cells were seeded in RPMI-1640 containing 10% FBS and treated with 0 (control) and 20 μg/mL of TBWSP31 for 48 h. After incubation, cells were collected and fixed in 2.5% (V/V) glutaraldehyde at 4 °C for SEM analysis [29]. Fixed cells were rinsed in 0.1 M phosphate buffer, pH 7.2 and post-fixed with 1% osmium tetroxide. Cells were rinsed in phosphate buffer and dehydrated in a graded ethanol series of 30, 50, 70, 90, 100% for 20 min. Cells were then critical point-dried from CO_2_ in a critical point dryer (CPD-030, BAL-TEC Co.). The dried cells were affixed to an aluminum stub with double-stick tape, coated with gold in an ionic sputter coater (SCD-005, BAL-TEC Co.). They were viewed and photographed by scanning electronic microscopy (QUANTA-200, FEI CO.).

### 3.7. Quantitative Detection of Cell DNA with Flow Cytometry (FCM)

Bcap37 cells were seeded in RPMI-1640 containing 10% FBS and treated with 0 (control), 5 and 10 μg/mL of TBWSP31 for 24 h and 48 h. Cell cycle analysis was performed using a hypotonic solution of propidium iodide (PI). Approximate 1 × 10^6^ cells per experimental condition were harvested, washed with cold PBS twice and fixed with 70% ice-cold ethanol at −20 °C. After fixation, cells were washed twice with PBS, then incubated with 100 μg/mL RNase A for 30 min at 37 °C and stained with 100 μg/mL propidium iodide (Sigma Chemical Co) for 30 min at room temperature. DNA fluorescence was measured in a Becton Dickinson flow cytometer, and the percentages of cells in G_0_/G_1_, S, and G_2_/M phases of the cell cycle were determined using the CellFIT software.

### 3.8. Determination of the Expression of bcl-2 and Fas

Bcap37 cells were seeded in RPMI-1640 containing 10% FBS and treated with 0 (control), 5 and 10 μg/mL of TBWSP31 for 48 h. After incubation, cells were fixed with 4% paraformaldehyde at room temperature for 30 min, washed with PBS, and then permeabilized with 0.2% Triton X-100. The cells were then incubated in PBS with either bcl-2 or Fas for 30 min at 37 °C. After two washes, the cells were incubated in PBS with the secondary FITC-conjugated goat anti-rat IgG for an additional 30 min at 4 °C in the dark. Subsequently, the cells were washed twice with PBS and analyzed by flow cytometry.

### 3.9. Statistical Analysis

Statistical analyses were performed using the SAS 8.1 software package (SAS Institute, Cary, North Carolina). Statistical significance was determined by analysis of variance (ANOVA) (*p* < 0.05).

## 4. Conclusions

In this work we investigated the antitumor mechanism of TBWSP31 on the human breast cancer cell line Bcap37 by inverted microscopy, scanning electron microscopy and flow cytometry. Typical apoptotic morphological changes were observed after incubation of Bcap37 cells with TBWSP31, such as detachment from the culture plate, no obvious microvilli, plasma membrane blebbing, and formation of apoptotic bodies. Cell-cycle analysis revealed that treatment with TBWSP31 resulted in G_0_/G_1_ arrest and prevented the cells from growing from G_0_/G_1_ phase to S phase. The expression of bcl-2 and Fas were detected quantitatively by FCM, which showed that TBWSP31 induced-apoptosis may be involved with up-regulation of Fas expression and down-regulation of bcl-2 expression. From the above facts, we conclude that TBWSP31 could inhibit the growth of Bcap37 cells via induction of apoptosis.

## Figures and Tables

**Table 1.** Effect of TBWSP31 treatment on the Bcap37 cell cycle.

**Time (h)**	**Concentration (μg/mL)**	**G_0_/G_1_ (%)**	**S (%)**	**G_2_/M (%)**
24	control	53.6	33.5	12.9
	5	57.3	31.4	11.3
	10	68.4	28.9	2.7
48	control	54.9	32.7	12.4
	5	70.9	28.5	0.6
	10	77.6	21.8	0.7
